# Some Thoughts on the Therapeutics of Parturition

**Published:** 1878-11

**Authors:** S. C. Dumm

**Affiliations:** Constantia, Ohio


### SOME THOUGHTS ON THE THERAPEUTICS OF PARTURITION.

---

By S. C. DUMM, M.D., Constantia, Ohio.

---

Cases where there are inefficient pains in some stage of labor, and especially the first stage, I am in the habit of treating with uterine stimulants and relaxing remedies, such as warm teas, bathing feet in hot water, sitz baths, warm vaginal injections, whisky, quinine, ergot, external manipulation, change of posture, rupture of the membranes, etc.

When called early to a case of labor, before there is any dilatation, with irregular, lancinating pains, I prescribe about one-eighth or one-sixth of a grain of morphia. If the pains are false this will generally quiet them down. But if labor proceeds, it regulates the pains and keeps them from returning so often, but increases their severity. I regard morphia in stimulating doses as one of our most useful uterine stimulants. It seems also to have a relaxing effect upon the circular fibres of the os, and in turn stimulates the longitudinal fibres of the body to stronger and more protracted contractions. Hence, I seldom fail to give it in those cases characterized by rigidity. It should be given with discrimination, however, for if we get too much it weakens the pains; but I have not hesitated to give it in full doses where the labor was protracted and the patient exhausted, to quiet down the pains and let the patient rest for an hour or two. I have noticed that they would waken up refreshed, and labor would go on with greater energy to successful termination.

I remember to have had one case of abortion at five months, where the patient suffered so severely with the pains that I gave full and repeated doses to restrain them; but to my surprise it acted just like ergot, and the pains were continuous for the next eight or ten hours, or until the partially decomposed fetus was expelled.

In a great many instances, hot pepper or ginger teas answer all purposes as a uterine stimulant, and these simple though efficient remedies should not be ignored.

In many cases whisky or brandy has been, in my hands, a satisfactory agent; but it is a remedy I never use myself, and I dislike to prescribe it very much; when given, it should be given in small quantities and often.

Ergot has, in my hands, proved perfectly satisfactory as a uterine stimulant, and, what may seem strange to some, I have not hesitated to prescribe it in every stage of labor. In the beginning of labor, where there is a lax condition of the os with inertia, or even where there is slight rigidity, I have given it in small doses with satisfaction, having never had any ill effects. Where there are strong pains, with rigidity of the os, I think it a dangerous remedy, and in such cases should not be given unless it be in the few last expulsive pains of the second or third stages of labor. But I find that authors are unable to lay down directions for its administration, and a practitioner must be guided by his own judgment, and his success will be according to his intuitive knowledge of what his patient will bear. When it is given in the first stage of labor it should be given in very small doses, and its effects carefully watched.

Where vaginal injections are used to promote relaxation, they should be composed of some mucilaginous fluid, as flax seed tea, slippery elm, etc. In using simply hot water it removes too much the natural lubricating mucus, which is poured out in great abundance, and thus leaves the parts hot and dry, and so retards the labor.

Where there is too great a quantity of liquor amnii, the walls of the uterus, being stretched to their utmost capacity, will not contract with any degree of force. Under such circumstances it is necessary, in order to expedite labor, to rupture the membranes, and by thus draining off the superabundant fluid, the uterus is enabled to concentrate its powers. I regard this as a valuable procedure, and have had recourse to it many times with great satisfaction.

I never treat a case without recourse to external manipulation, making pressure with the left hand by grasping, or rather kneading, the uterus, commencing with the pain. In the beginning of the second stage, where the head of the child is large and the woman muscular, it does not advance as it should. Then we may place the woman upon her left side, and make pressure with the left hand over the uterus, while we introduce the fingers of the right up well, and lift the perineum as with Sims' speculum; this procedure will let the head advance rapidly in many cases.

Where there is delay in the head passing the vulva, by introducing one or two fingers into the rectum and making pressure, we may assist labor materially.

I have often noticed that in many cases there is an obliquity of the uterus, more especially a tipping forward of the fundus. In such cases the head of the child presses against the anterior lip of the uterus, and hinders advancement. In such cases I hook one finger into the os, between pains, and draw it forward, holding it up until another pain comes and establishes its natural position.

I never allow the placenta to remain over fifteen or twenty minutes. I generally give a dose of fluid extract of ergot, and practice "Crede's method," which consists in grasping the uterus through the wall of the abdomen and squeezing out the placenta, holding on to the cord and pulling it gently.

I have used chloroform in a few cases, pouring a small quantity upon a napkin, and letting the patient take two or three inspirations at the beginning of a pain. This is sufficient to ease the pain somewhat without lessening its force.

I have never had but one severe case of flooding, and I attribute my success in this particular to the external manipulation made use of in labor before referred to, thus keeping up continuous contraction. This case was treated by lowering the head, ergot, cold water to the abdomen, with stimulants. The case was a severe one, but the treatment proved satisfactory.

In cases of abortion, if the pains are insufficient, and placenta retained with hemorrhage, I use ergot, and hot water injections up to the os. These should be used just as hot as the patient can bear. They will generally bring on pain, check the hemorrhage and expel the placenta.

I had one case, however, where nothing seemed to give any relief, and with all my efforts, I could not get the placenta away. It sloughed away in three or four weeks, with occasional hemorrhages, which were controlled with the tampon.

In puerperal fever, I have used with success hot water injections, or a weak solution of carbolic acid, every three or four hours. This seems to give great relief to pain and soreness, and also washes away unhealthy discharges. It also acts somewhat as a poultice, inviting the flow of lochia. This treatment, with fomentations externally and opium and quinine internally, has been the treatment most successful in my hands.

